# Aptitude measurement: is measurement validity compromised in the morning

**DOI:** 10.3389/fpsyg.2023.1210958

**Published:** 2023-08-22

**Authors:** Georgios Sideridis, Fathima Jaffari

**Affiliations:** ^1^Boston Children’s Hospital and Harvard Medical School, Boston, MA, United States; ^2^Department of Research, National and Kapodistrian University of Athens, Athens, Greece; ^3^Education and Training Evaluation Commission, Riyadh, Saudi Arabia

**Keywords:** morning evening testing, chronotypes, achievement, measurement invariance, construct reliability and validity, aptitude

## Abstract

The purpose of the present study was to evaluate the reliability and validity of the General Aptitude Test (GAT), a national instrument for the measurement of aptitude/achievement in the Kingdom of Saudi Arabia as a function of daytime testing. Participants were 722 students who took on the GAT across morning and evening administrations in a within-person pre-post design. Participants were matched for gender, parental education, and test center characteristics (i.e., size). The GAT was tested for its psychometric properties and its measurement invariance across time of day. Results pointed to a significant misfit using an exact invariance protocol. Specifically, there was a large number of non-invariant items pointing to Differential Item Functioning (DIF). Second, internal consistency reliabilities were consistently lower during morning testing compared to evening testing as evidenced using both statistical and visual means. Concerns about dimensionality were also raised for the morning compared to the evening administration. Last, comparison of performance levels indicated that morning testing was associated with significant decrements in performance across all domains compared to performance levels during evening testing. The results have implications for the validity of measurement and public testing policy if test validity during morning administration is compromised.

## 1. Introduction

The examination of a person’s natural ability or innate talents in a certain area of functioning is what is meant by the term “aptitude measurement.” It is common practice in educational and employment environments to use aptitude tests as a means of evaluating an individual’s potential for learning, their level of motivation, the tactics they use to solve problems, and their level of performance on particular topics (Kuncel et al., 2004). These assessments can assist determine a person’s strong and weak points, as well as shed light on whether or not they would be a good fit for a certain course of study (such as one that would lead to admittance to a higher education institution) or lead to an offer of employment (Cronbach and Snow, 1977). As a result of the fact that aptitude tests are a factor in the formation of well-informed judgments regarding an individual’s potential and, as a consequence, their success in education or job, the reliability and validity of these assessments becomes of the utmost importance.

College education in particular is regarded as the most essential step of a student’s educational journey because it is the primary key to entering the labor market and finding a job that is fit for one’s skills and interests. The performance of high school graduates on pre-college assessments of aptitude, which are officially employed as one of the criteria for university entrance, is one of the factors that determine whether or not they will be admitted to a good college program (Sackett et al., 2016). This factor is just one of several. The General Aptitude Test is a standardized pre-college ability test that is used for admission to colleges in Saudi Arabia and eligibility decisions are based on its results.

An individual’s performance on an aptitude test can be influenced by a wide variety of factors. The level of an individual’s motivation, level of knowledge of the content being assessed, level of confidence, level of experience, anxiety, exhaustion, and overall health are all examples of individual dispositions (e.g., Credé et al., 2017; Ackerman, 2018). An individual’s performance on an aptitude test may also be impacted by environmental elements, such as the presence of interruptions, diversions, or the characteristics of the testing site. We predict that testing in the morning vs. testing in the evening may be associated with various levels of psychometric quality measures, hence the purpose of the current study is to investigate the function that time of day plays in the measurement process. The following line of reasoning, which relates to climate and cultural factors in the Kingdom of Saudi Arabia, serves as the foundation for this argument.

A person’s natural predisposition to feel more attentive and productive at a given time of the day may be the source of their preference for engaging in academic work either in the morning or in the evening. This tendency is also known as a person’s chronotype, and it is determined by an individual’s circadian rhythm, also known as their biological clock. It is manifested by increased alertness and energy at a specific time, which most likely affects not only performance and productivity but also overall well-being and health. According to this conception, it is essential for people to be aware of their chronotypes and to organize their work schedules and working environments in ways that complement their natural cycles of sleeping and being awake. The empirical research has not produced anything even close to conclusive results. Enright and Refinetti (2017) conducted a study using a sample of university students who took classes and exams at different times of the day to investigate the effect that the time of testing has on the performance of students on standardized tests. The students were required to take the tests at the same time each day. This finding is consistent with findings from earlier empirical research, and the outcomes of this study demonstrated that morning students had a higher performance level than evening students on the tests (e.g., Hartley and Nicholls, 2008). Piffer et al. (2014) discovered that evening participants scored higher on the GMAT than morning students did, and this was true independent of gender.

In Saudi Arabia, aptitude tests can be given in the morning or the evening, depending on the candidate’s schedule and personal inclination. Some people, for instance, prefer testing in the morning because they may feel more alert and rested at that time of day. Other people, however, favor testing in the evening (or any other time of day, for that matter) because they are free of obligations, they are less likely to be disrupted, and that fits with their particular schedule and way of life. The empirical data collected in the Kingdom have indicated that the vast majority of participants (95%) would prefer to take the test in the evening.

Specifically, there is evidence that the quality of sleep is poor in Saudi Arabia (AlRasheed et al., 2022), particularly during the covid pandemic (Iqbal et al., 2021) for university students as well as elementary school children (BaHammam et al., 2006). According to a recent study in the Kingdom, “sleep duration was short, which potentially has significant implications in general public safety, productivity, and quality of life” (AlAhmari and Alshehri, 2019, p. 144). According to Hakami et al. (2021), such poor sleeping patterns in Saudi Arabia were connected with sluggishness, exhaustion, lack of focus, and an inability to be awake and wakeful when attending school. These findings were supported by Zeb et al. (2020), who found that college students who had poor sleep habits finished fewer tasks, were less aware, and received negative ratings on attention from faculty input. It is interesting to note that the average amount of sleep that college students get around the world ranges anywhere from 6.4 to 8 h per night (e.g., Sweileh et al., 2011). It is also interesting to note that estimates for Saudi college students are exactly at the low bound estimate of 6.4 h that is reported in the international literature (Ahmed et al., 2017). Al-Hazzaa et al. (2012) indicated that similar quantities were found in Saudi Arabian adolescents between the ages of 15 and 19 years old. Last but not least, these findings were also replicated with a sample of medical students, in which sleep deprivation was connected to poor academic achievements (Abdulghani et al., 2012; Alhusseini Ramadan et al., 2022).

There are questions about whether these results would hold true in the Kingdom of Saudi Arabia despite the overwhelming empirical evidence in favor of morning testing in the international literature, which is partly driven by a “circadian preference for daily activities” (Putilov, 2017). According to Önder (2022), sleep deprivation and the presence of light pollution alone cause a mismatch between the biological and social rhythm when a late-night nap is taken when an early wake-up is necessary (Ahmed et al., 2017). Consequently, for students in Saudi Arabia early class attendance and morning testing may suffer for reasons discussed next. Disrupted sleep is associated with excessive daytime sleepiness if an early wake-up is necessary (AlAhmari and Alshehri, 2022). According to current perceptions in the Saudi Arabian Kingdom, staying up late is a common practice for most Saudis and has integrated into the country’s purported culture (Al-Ajlouni, 2019; Mirghani et al., 2019). The heat during the day inhibits carrying out errands and other duties obstructing focus and concentration, delaying them until evening and nighttime, and the lengthy prayer hours also interrupt extended and focused working habits. These are the two main explanations given. Given these conditions, it goes without saying that students—especially those in higher education—study late. It remains to be explored how these late-night routines affect slumber and academic activities. So, despite the fact that there is a wealth of research in western countries that supports early morning engagement and academic performance, it is intriguing to consider how these results apply to the assessment of aptitude among Saudi high school students. The main hypothesis of the present study was that morning testing would be linked to enhanced measurement error of the GAT as evidenced using indicators of reliability and validity.

## 2. Method

### 2.1. Participants and procedures

The sample consisted of 722 high school students who took the Graduate Aptitude Test- GAT-for science majors in March 2022 (196 males, 27.1.%) and (526 females, 72.9%). The mean age was 17 years and 6 months with a standard deviation of 11 months. The age range was 11 years (minimum 17 to maximum of 28 years). The data were collected from the Educational Testing and Evaluation Commission (ETEC) and the Ministry of Education of the Saudi Arabia Kingdom. Information about gender, parental education, test center location, and the size of the facilities was utilized to create matched groups. Data were checked for entry errors and omissions and it was further confirmed that there were no missing values for any participant in any domain and hence no treatment for missing data.

To ensure the time of testing group equivalence a propensity score matching protocol was applied to account for potential differences in gender, and parental education given the preference for evening testing. Thus, all morning testing participants were matched with evening testing participants using optimal matching (Hansen, 2004). All analyses were run using the MatchIt package in R (Ho et al., 2011). Post-matching tests indicated no differences between morning and evening groups on gender mother’s education, father’s education, and test center facility size. Exclusionary criteria were participant non-response and early withdrawal from the testing facility, or documented cheating, rending the assessments invalid. The sample size was guided by the availability of morning testing as all morning participants were utilized and were matched to evening participants. The project was approved by the ethics committee in ETEC on August 15, 2022.

### 2.2. Measures

#### 2.2.1. Graduate aptitude test for science major (GAT-Science)

The test was divided into two components: verbal and quantitative with a total number 96 items distributed differently on the two parts; the verbal part includes 52 items representing four sections: verbal Analogy (16 items), sentence Completion (10 items), synonymy (6 items), and reading comprehension (20 items). The quantitative part contains 44 items representing five sections: arithmetic (16 items), geometry (8 items), algebra (4 items), data analysis (8 items), and comparison (8 items). All items utilize a dichotomous scaling format (0/1).

### 2.3. Data analyses

#### 2.3.1. Item factor analysis

Data were analyzed using an item factor analysis with dichotomous indicators. Initial tests involved testing various models to optimally define the simple structure of the instrument (tests of factorial validity). The comparative models involved a unidimensional structure, a two-factor correlated structure, a 9-factor domain-specific structure, and a bifactor model. After concluding the optimal factor structure a measurement and structural invariance protocol was employed to verify the equivalence of form and function across time of testing occasions as described below. First, the configural model was tested which evaluated the equivalence in form across the time of testing. This was followed by the metric model in which the relationships between items and latent variables were tested. The third model, which represents a prerequisite before contrasting means was the scalar model specifying, in addition to the configural and metric models, the equivalence of intercept terms. If any of the measurement invariance tests fail, we will employ additional means to satisfy invariance so that latent mean testing can be further pursued. To this end, we will employ the alignment methodology (Asparouhov and Muthén, 2014) using fixed alignment as has been recommended when only two groups are present. For the procedure to be successful only minimum non-invariance should be present among estimated parameters. The method engages the configural model and then employs the simplicity function (Muthén and Asparouhov, 2018) to minimize non-invariance in factor loadings and intercept terms.

#### 2.3.2. Reliability and unidimensionality

We used Cronbach’s alpha, and McDonald’s Omega as our evaluative criteria for internal consistency reliability [for more information the reader is directed to the works of Cronbach (1951) and Raykov (1997)]. For unidimensionality, we employed two analytical means, namely estimation of the DETECT index (Dimensionality Evaluation to Enumerate Contributing Traits) and by visualizing Cronbach-Mesbach curves (Mesbah, 2010). A brief description of the two follows next.

The DETECT index (Zhang and Stout, 1999a,b) was developed to evaluate essential dimensionality (Nandakumar, 1991; Monahan et al., 2007). This analytical method involves partitioning the items into clusters so that within cluster homogeneity is maximized and between clusters are separated. Studies have shown that the index is biased with small samples or brief scales (Monahan et al., 2007). Under perfect unidimensionality the index has an expected value of zero. Conventions suggest that estimates between 0.1 and 0.5 are indicative of weak multidimensionality, estimates between 0.5 and 1 are indicative of moderate multidimensionality and estimates greater than 1 of strong multidimensionality (Nandakumar and Stout, 1993).

The Mesbah curve utilizes Cronbach’s alpha as a means to evaluate first-factor saturation. The figure displays an increasing curve (reflecting increases in alpha) as a function of increases in the number of items. Under these lenses, if a measure is multidimensional, the coefficient decreases with the inclusion of items that no longer contribute to the first dimension, thus, reflecting a quadratic-type curve. This step-by-step procedure results in a curve that identifies the items that contribute stochastic information to the latent trait.

#### 2.3.3. Item misfit

Several procedures were involved to evaluate item misfit. Specifically, we engaged estimates of correlated residuals using the LD Chi-square test, and the chi-square test that evaluates Guttman-like patterns. All analyses were conducted using Mplus 8.10 (Muthén and Muthén, 1998–2022), IRTPro, JAMOVI, DIMPACK, and the R package CMC.

## 3. Results

### 3.1. Factorial validity of the GAT using confirmatory factor analysis

Table 1 displays model fit statistics of the GAT using 4 competing models. Model 1 (M1) is unidimensional and served as a reference comparison model as it deviated from model theses. Model 2 (M2) describes a 2-correlated factor model testing the hypotheses of two major domains, namely verbal and mathematics, collapsing all subdomains within each of the two main domains. Model 3 (M3) tests a 9-factor model structure with each verbal and quantitative domain being modeled as a separate entity. Last, Model 4 (M4) presents a bifactor model with items loading on both a general factor and also the 9 domain-specific factors. The use of inferential statistical criteria was precluded as models were not nested. Instead, we relied on using information criteria and specifically the Bayesian Information Criterion (BIC).

**Table 1:** Model fit for GAT science using item factor analysis (IFA) and tests of measurement invariance across time of day.

Model	Chi-square	D.F.	CFI	TLI	RMSEA	Model comparison	ΔChi-square	ΔD.F.	Value of *p*
Tests of model fit									
M1. Unidimensional	5256.455***	4,464	0.942	0.940	0.016	–	–	–	–
M2. 2-Factor correlated	5150.172***	4,463	0.949	0.948	0.015	–	–	–	–
M3. 9-Factor correlated	5073.323***	4,428	0.952	0.951	0.014	–	–	–	–
M4. Bifactor model	5009.890***	4,368	0.953	0.951	0.014	–	–	–	–
Tests of measurement invariance across time-of-day occasions									
M3a. Configural model	9161.623***	8,856	0.974	0.973	0.010	–	–	–	–
M3b. Metric model	9674.065***	8,943	0.937	0.935	0.015	M3a vs. M3b	251.462***	87	<0.001
M3c. Scalar model	9929.188***	9,030	0.922	0.921	0.017	M3b vs. M3c	655.537***	87	<0.001

***p < 0.001. CFI, comparative fit index; TLI, Tucker–Lewis Index; RMSEA, root mean square error of approximation; ΔChi-square, difference chi-square estimate across competing models; ΔD.F., difference in degrees of freedom; value of *p*, probability estimate of difference chi-square test. Model comparison not contacted using inferential criteria because models differ in structure and thus are not nested.

The first observation was that the bifactor model, regardless of fit, was uninterpretable as no domain-specific factor “survived,” thus domain specificity for at least some domains was not supported. Consequently, we focused on the remaining model tests. The smallest BIC, besides the bifactor model was for the 9-factor correlated model (BIC = 5073.169) followed by the 2-factor model (BIC = 5150.019) and last the unidimensional model (BIC = 5256.302). The 9-factor model had all descriptive fit indices (i.e., CFI, TLI) over 0.95, and unstandardized residual values less than 2%, being indicative of “exact model fit” as per the MacCallum et al. (1996) recommendations. Further tests of measurement invariance utilized M3 as reflecting the optimal simple structure.

### 3.2. Measurement invariance across time of testing using CFA

Measurement invariance across the time of testing is shown in the bottom part of Table 1. Initially, a configural model was fitted to the data, which showed an acceptable model fit in that the 9-factor correlated simple structure fit the data well at both time-of-day occasions. Further testing involved more constrained models as a means to examine the decrease in fit due to non-invariance. The metric model specified the equivalence of factor loadings across time-of-day testing with thresholds being freely estimated. Results indicated that constraining the slopes to be equivalent across measurement occasions was associated with significant decrements in model fit [Difftest(87) = 251.462, *p* < 0.001]. The scalar model involved the additional constraint of the equivalence of thresholds across time instances in addition to the equivalence of slopes (although the metric model did not hold). The scalar model was again pointed to a significantly worse model with the imposition of equal thresholds across time points [Difftest(87) = 655.3537 *p* < 0.001] following a misspecified metric model.

An in-depth analysis of the item level behavior across measurement instances is shown in Table 2 with the inclusion of indices of Differential Threshold Functioning (DTF), tests of correlated residuals, and item misfit using chi-square tests. Our choice of DTF is based on the fact that item response theory and confirmatory factor analysis (CFA) are complementary approaches when focusing on scale development (Bean and Bowen, 2021), thus, Differential Item Functioning (DIF) in IRT is largely equivalent to the equivalence of thresholds across groups in the CFA format. As shown in the table, non-invariance was non-negligible; That is, DTF was observed in 44 items out of 96, representing 45.8% of the items. Given that domain VSC had no DTF items, MGE only 1 DTF item, and MAL two items, it is apparent that for the remaining domains, the majority of the items exhibited significant DTF. These results cast doubt on the functioning of the instrument over time with serious implications for the measurement of GAT. Results concerning local dependency and correlated content between items within a domain indicated a few significant effects as well: there were 3 correlated residuals in the morning and 1 in the evening testing occasions for domains VRC, MAR (in the morning), and VAN (in the evening) suggesting minimal content overlap or the presence of a third variable content that governs the items. Last, item misfit using the chi-square test indicated 21 misfitted items in the morning measurement and 22 during the evening, showing largely speaking equivalence using that criterion.

**Table 2:** Item and domain quality criteria of GAT across measurement occasions.

GAT		No of items with local dependency		No of items with misfitted chi-square		Cronbach alpha		Omega reliability		Detect value	
CConstruct	DTF items	Morning	Evening	Morning	Evening	Morning	Evening	Morning	Evening	Morning	Evening
1. VAN	i3, i4, i5, i6, i7, i9, i11, i13, i15	0	1	3	6	0.71	0.80	0.72	0.80	0.185	0.079
2. VCA	i2, i3, i7	0	0	2	0	0.68	0.69	0.68	0.70	0.292	0.150
3. VSC	None	0	0	3	0	0.48	0.56	0.49	0.57	0.719	0.434
4. VRC	i1, i2, i4, i5, i8, i9, i10, i12, i13, i14, i18, i20	1	0	2	0	0.46	0.70	0.46	0.71	0.306	0.078
5. MAR	i3, i6, i10, i11, i12, i13, i14, i15	2	0	5	1	0.51	0.74	0.52	0.75	0.364	0.189
6. MGE	i7	0	0	1	2	0.39	0.52	0.39	0.52	0.646	0.593
7. MAN	i1, i3, i4, i5	0	0	3	1	0.35	0.61	0.35	0.62	0.860	0.359
8. MAL	i1, i2	0	0	0	4	0.27	0.33	0.30	0.46	1.647	1.494
9. MCO	i1, i2, i3, i4, i6	0	0	2	8	0.54	0.53	0.55	0.54	0.530	0.770

**Figure 1:**
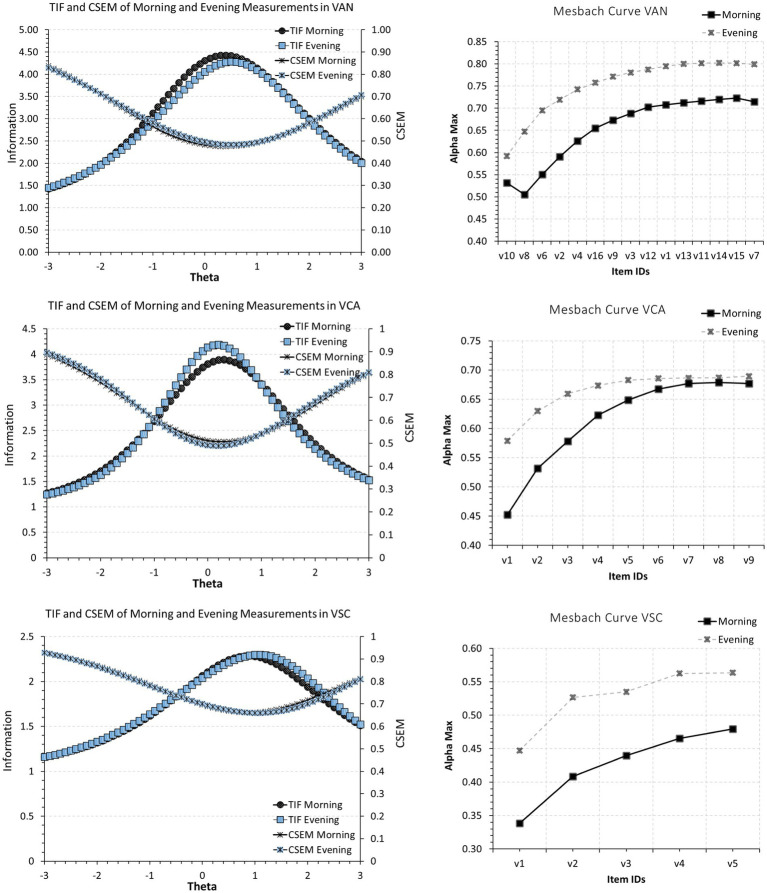

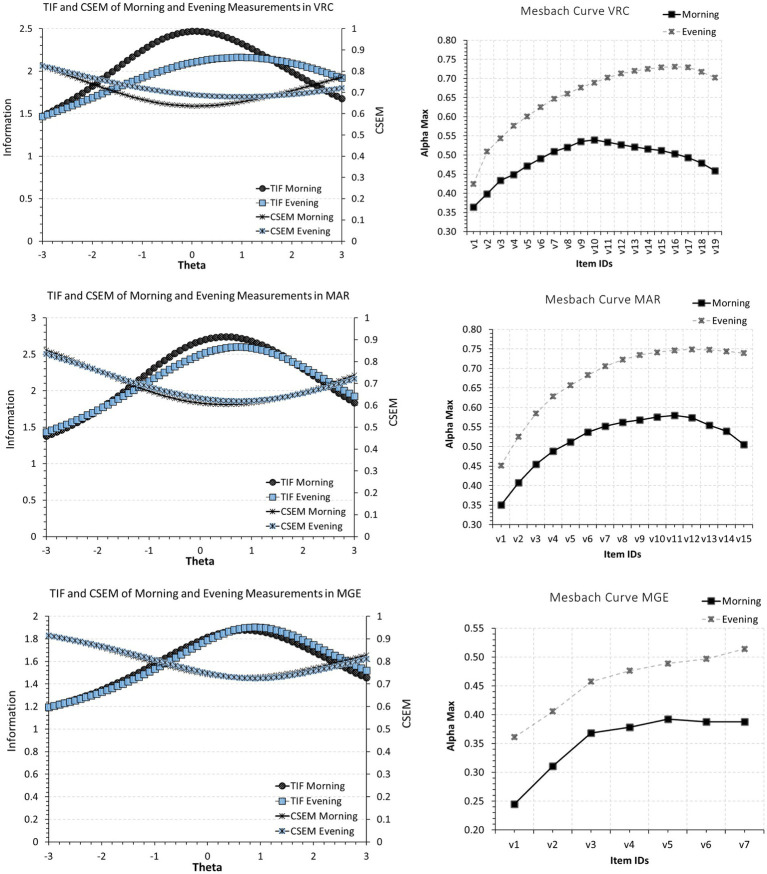

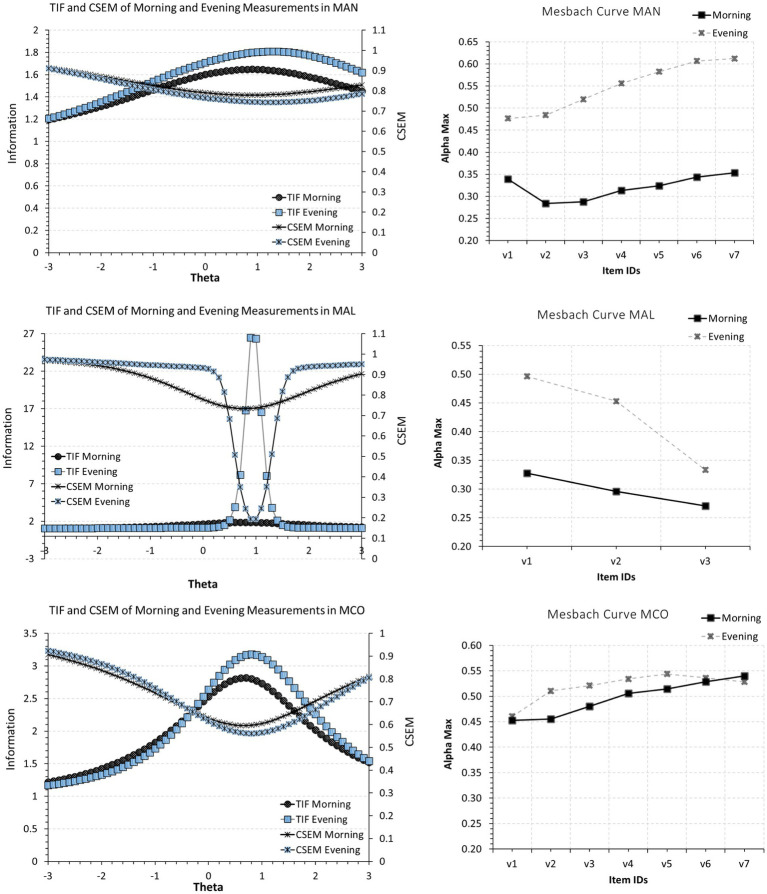
Scale information functions for GAT across morning and evening administrations.

A last indicator of non-equivalence is provided by plotting domain information functions across measurement occasions. As shown in Figure 1, there are visually speaking discrepancies between information functions over time but also substantial overlap. For example, the domains VRC, MAR, MAN, and MCO showed a trend for a higher sensitivity to increased levels of theta during the evening compared to morning testing with approximately the same amount of information. All other domains had approximately equal sensitivity to theta levels across morning and evening testing.

### 3.3. Reliability and unidimensionality

Following the failure to satisfy measurement invariance, additional tests were implemented to evaluate differences across the instrument over time. Table 2 displays internal consistency reliability estimates across measurement points using three indices of reliability namely, Cronbach’s alpha, Omega, and Marginal reliability. As shown in the Table, out of 27 comparisons results indicated that using visual analysis’s reliability during morning testing was lower compared to that during evening testing in 26/27 comparisons (again not utilizing inferential statistical means). Collectively these results point to the non-equivalence between measurement occasions.

### 3.4. Latent mean comparisons across time of testing occasions

**Figure 2:**
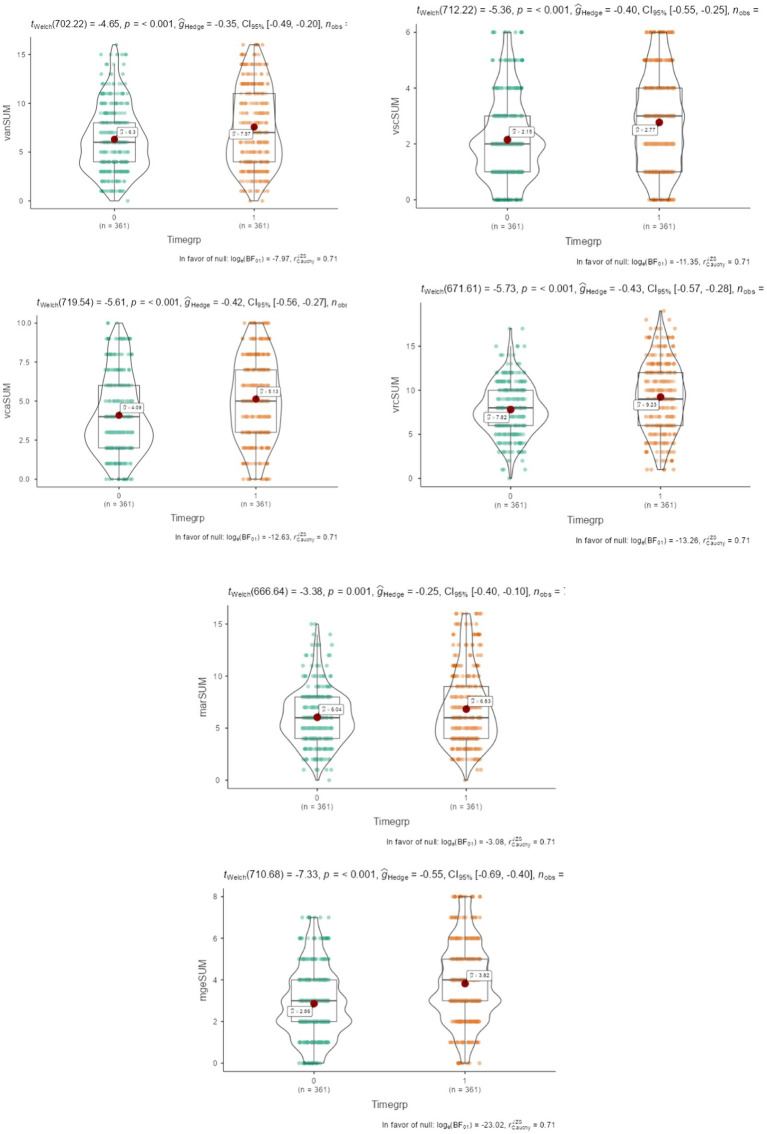

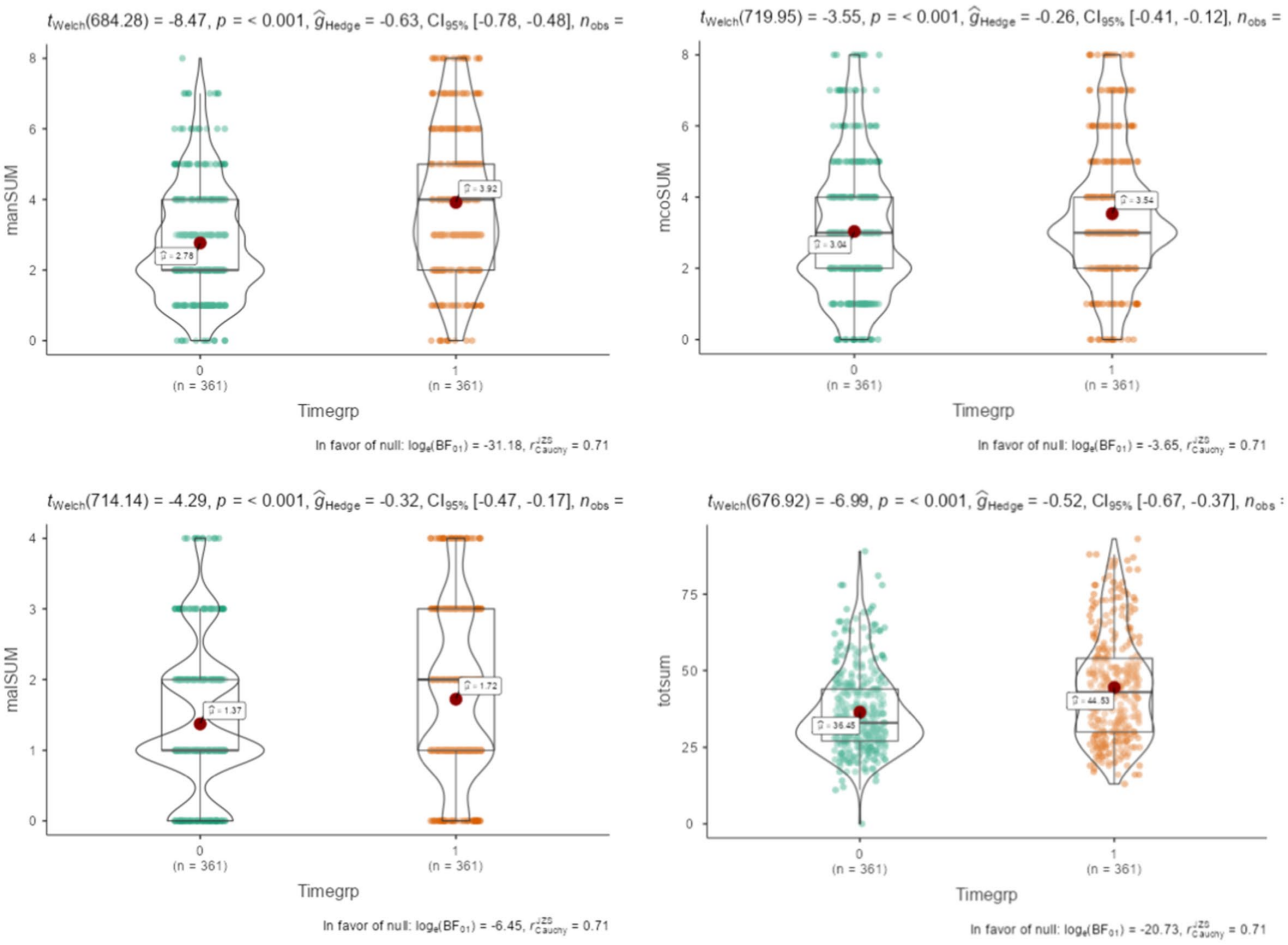
Violin plots for comparisons of means across measurement occasions. The left plots reflect morning measurements and the right plots evening. Tests of significance with a negative sign favor evening measurement.

To overcome the problem of non-invariance with the goal of contrasting means, the alignment procedure was engaged. The goal was to identify possible trends in mean levels as a function of the time of testing. Figure 2 displays these results with violin plots, tests of significance, and Hedges g effect size estimates. Results using t-tests indicated that across all domains, evening testing was associated with higher performance compared to morning testing. Effect sizes ranged between −0.25 and −0.63 standard deviations being in the range of small, small to medium, medium, and medium to large (Cohen, 1992), but none exceeded large effect sizes.

## 4. Discussion

The purpose of the present study was to evaluate the construct validity of the GAT, a national instrument for the measurement of aptitude/achievement in the Kingdom of Saudi Arabia as a function of daytime testing. Several important findings emerged.

The most significant finding was that there was no consistency in the results obtained in the morning and evening tests. According to the findings of measurements’ consistency tests, the instrument carried out its functions differently in the morning compared to the evening. The fact that over 46 percent of the items displayed significant DIF indicates that there are notable levels of non-equivalence. In connection with this first point, the findings of the morning tests revealed consistently lower levels of internal consistency reliability than those of the evening tests. These findings raise concerns regarding the reliability and validity of the measurements taken in the morning tests. Apparently, increased amounts of systematic measurement error lowers expectations on score accuracy, and consistency of the observed levels over time. The GAT very strongly suggested that there was no equivalence, even though the reasons for this non-equivalence were not addressed in the present cross-sectional design.

After reaching a certain minimum degree of invariance, a second notable finding was that morning testing was associated with significantly lower achievement levels. This finding agrees with the work of Alhola and Polo-Kantola (2007) who highlighted the moderating role of sleep deprivation. A later wake-up time was also linked to better academic performance, according to Sofyana et al. (2022), since it mitigates the impacts of sleep deprivation with the negative effects of the latter being more pronounced in males (see also Pilcher et al., 1997; Watson et al., 2015; Ohayon et al., 2017; Ross et al., 2020). For Saudi university students, Buragadda and Al-Eisa (2016) reported higher levels of academic performance during the evening compared to the morning and a preference for evening lateness, which eventually can lead to Delayed Sleep Phase Disorder (DSPD). The above empirical studies’ findings agree with the present results after reaching partial measurement invariance. Several thoughts are in order regarding this finding, albeit at the speculative level. First, due to the heat, people may be more accustomed to engaging in intellectual activities in the evening. If that’s the case, then the operation of circadian rhythms (Carrier and Monk, 2000) likely regulates physiological processes so that a state of wakefulness is more present in the evening hours compared to the morning (Harder-Lauridsen et al., 2017). Second, the shift in social and recreational activities later in the day may result in late night sleeping that comparatively affects energy levels in the morning, especially as individuals have to accommodate early praying in the morning, resulting in sleepiness and fatigue in the early morning hours (Pilcher and Huffcutt, 1996; BaHammam et al., 2012).

The present findings are in line with the suspected culture of night work/recreation which has been integrated into the country’s functioning (Al-Ajlouni, 2019 Mirghani et al., 2019). The poor academic performance during morning testing observed in the present study is likely linked to the causal mechanism of sleep deprivation and circadian disruption (McEwen and Karatsoreos, 2022), which is manifested with decreased motivation, impaired memory, difficulties concentrating (Wolfson and Carskadon, 1998; Dewald et al., 2010) or cognitive recovery (Alhola and Polo-Kantola, 2007) as well as decreased physical and mental health (Gruba et al., 2021; Griggs et al., 2022). Practically speaking, the government might launch campaigns to raise public knowledge of healthy sleeping practices and the hazards of sleep deprivation to one’s health. Such initiatives could be expanded to communities, schools, and public health organizations. The government can also adjust workplace and school timing policies to ensure students and workers receive adequate sleep. The present study is limited for several reasons. First, the study design is correlational and thus causal inferences cannot be made. It is possible that other third variables and confounders were responsible for the present trend. Second, the sample size was modest, and it is possible that if morning testing was preferred by more participants, the results may have been different. In the future, it will be essential to determine whether or not the non-equivalence may be attributed, at least in part, to the test center as well as the various circumstances during test taking.

## Data availability statement

The raw data supporting the conclusions of this article will be made available by the authors, without undue reservation.

## Ethics statement

The studies involving human participants were reviewed and approved by ETEC. The patients/participants provided their written informed consent to participate in this study.

## Author contributions

GS conceptualized the study and contributed to data analyses and the write-up of the manuscript. FJ contributed to data analyses and the write-up of the quantitative sections and also contributed the data for the present illustration. All authors contributed to the article and approved the submitted version.

## Funding

This project was funded by ETEC, Riyadh, Saudi Arabia. Approval of the project was provided by ETEC on August 15, 2022.

## Conflict of interest

The authors declare that the research was conducted in the absence of any commercial or financial relationships that could be construed as a potential conflict of interest.

## Publisher’s note

All claims expressed in this article are solely those of the authors and do not necessarily represent those of their affiliated organizations, or those of the publisher, the editors and the reviewers. Any product that may be evaluated in this article, or claim that may be made by its manufacturer, is not guaranteed or endorsed by the publisher.
